# A Novel Approach for Dynamic Testing of Total Hip Dislocation under Physiological Conditions

**DOI:** 10.1371/journal.pone.0145798

**Published:** 2015-12-30

**Authors:** Sven Herrmann, Daniel Kluess, Michael Kaehler, Robert Grawe, Roman Rachholz, Robert Souffrant, János Zierath, Rainer Bader, Christoph Woernle

**Affiliations:** 1 Department of Orthopaedics, University Medicine Rostock, Rostock, Germany; 2 Chair of Technical Dynamics, Faculty of Mechanical Engineering and Marine Technology, University of Rostock, Rostock, Germany; University of Zaragoza, SPAIN

## Abstract

Constant high rates of dislocation-related complications of total hip replacements (THRs) show that contributing factors like implant position and design, soft tissue condition and dynamics of physiological motions have not yet been fully understood. As in vivo measurements of excessive motions are not possible due to ethical objections, a comprehensive approach is proposed which is capable of testing THR stability under dynamic, reproducible and physiological conditions. The approach is based on a hardware-in-the-loop (HiL) simulation where a robotic physical setup interacts with a computational musculoskeletal model based on inverse dynamics. A major objective of this work was the validation of the HiL test system against in vivo data derived from patients with instrumented THRs. Moreover, the impact of certain test conditions, such as joint lubrication, implant position, load level in terms of body mass and removal of muscle structures, was evaluated within several HiL simulations. The outcomes for a normal sitting down and standing up maneuver revealed good agreement in trend and magnitude compared with in vivo measured hip joint forces. For a deep maneuver with femoral adduction, lubrication was shown to cause less friction torques than under dry conditions. Similarly, it could be demonstrated that less cup anteversion and inclination lead to earlier impingement in flexion motion including pelvic tilt for selected combinations of cup and stem positions. Reducing body mass did not influence impingement-free range of motion and dislocation behavior; however, higher resisting torques were observed under higher loads. Muscle removal emulating a posterior surgical approach indicated alterations in THR loading and the instability process in contrast to a reference case with intact musculature. Based on the presented data, it can be concluded that the HiL test system is able to reproduce comparable joint dynamics as present in THR patients.

## Introduction

Postoperative stability of total hip replacements (THRs) constitutes an essential objective to restore mobility and relieve pain for affected patients. Dislocation, however, remains a serious complications after total hip arthroplasty frequently leading to revision surgery. For example, 26% of all total hip revisions registered during a ten-year observation period in Sweden [1] were correlated to dislocation. Another study conducted in the United States [2] ranked dislocation even before aseptic loosening as major reason for revisions, with approximately 22% of 50,000 revision cases reported.

According to previous studies there are two plausible mechanisms for total hip dislocation. On the one hand, impingement events may occur with contact between implant components, leading to leverage of the femoral head. This component-to-component impingement determines the technical range of motion (RoM) of THRs for given design and positioning parameters, i.e. head size [3, 4], head-neck ratio [5], neck-to-shaft angle of the stem [6, 7], cup orientation [5, 8–10], and stem antetorsion [9]. Studies based on mechanical setups [11–13] or finite element models [4, 14, 15] analyzed THR stability under idealized load conditions. Their outcomes revealed that additional angular motion is required beyond the instant of first impingement before frank dislocation occurs. During this subluxation process the femoral head is levered out which is characterized by a resisting torque rising due to two contact points and dropping towards dislocation.

On the other hand, dynamic forces may separate the joint conjunction without previous impingement [15, 16]. Higa et al. [17] indicated spontaneous dislocation under passive conditions to occur at cup anteversion angles above 10° for high flexion movements combined with adduction and internal rotation. Other researchers introduced realistic motion data and compliant hip joint forces derived from a validated musculoskeletal model to simulate instability scenarios addressing both dislocation mechanisms [18]. Based on this approach, Pedersen et al. [19] illuminated Lewinnek et al.’s safe zone for cup placement [20] in view of activity dependent load cases. Comparable studies revealed declined dislocation resistance for increased lip radii of the liner owing to decreased head coverage [21] and elevated risk for obesity patients due to thigh-to-thigh contact induced spontaneous separation [22].

These findings suggest that the actual load situation plays a key role in the process of THR instability besides mere kinematic considerations. In the light of musculoskeletal dynamics, Heller et al. [23] revealed that muscle and hip joint loading may substantially change due to modification of the stem antetorsion. Similar results were reported by varying stem antetorsion as well as design parameters of the femoral component which also alter the location of the hip joint center with respect to the femoral bone [24–26]. Motivated by clinical investigations [27–29], researchers [30, 31] evaluated the effect of reattached muscular and capsular structures with respect to dislocation by using full-leg specimens. Their outcomes indicated enlarged resistance under repair. Likewise, Elkins et al. [32] exposed a dramatic loss in the resisting torque from an intact or well-repaired to a defected capsule for a sit-to-stand maneuver.

Despite such insights, there is still little evidence in how exactly active and passive soft tissue structures engage during total hip dislocation in patients. This is especially the case when implant design and positioning change the geometric proportions of the skeletal system and hence overall musculoskeletal dynamics. All studies and approaches quoted entail certain shortcomings, such that reliable and reproducible analyses are scarce meeting the requirements mentioned. Loading and motion were investigated for routine activities using instrumented implants [33], but in vivo testing of excessive load cases are ethically not possible.

Therefore, the purpose of this work was to present a comprehensive approach capable of reproducible testing of THR dislocation under dynamic and physiological conditions. The approach is based on a hardware-in-the-loop (HiL) simulation where a six-axis robot moves and loads a THR while bidirectionally interacting with a computational musculoskeletal model [34, 35]. Functionality of the physical setup [11, 34] and appropriate control modes for use in HiL simulations [36–38] were verified previously. Therefore, a major objective of the present work was to assess the HiL test system’s capability of delivering physiologically realistic test conditions. Another objective was to evaluate the impact of certain test conditions on the stability of the artificial hip joint for a representative dislocation-associated leg maneuver. These included joint lubrication, implant position, subject body mass, and removal of muscle structures emulating a posterior surgical approach.

## Methods

The HiL approach is first introduced by means of a functional principle. It describes the underlying interactions between the two integral components: the physical setup and the computational musculoskeletal model, both embedded into a control system. The real implant components are attached to the physical setup composed of a compliant support and an industrial robot running in hybrid position-force control. The model calculates muscle and reaction forces following inverse dynamics while incorporating implant design and positioning parameters. Required motion data is obtained by motion analyses with subsequent inverse kinematics of one healthy subject for two maneuvers. Based on the motion data, both model and physical setup were configured according to certain model and test parameters for carrying out several HiL simulations for the purposes of validation and parameter variation.

### Functional principle of HiL simulations for testing THR

The functional principle of the HiL approach is based on complementary sets of free and constrained directions of the artificial joint [36, 38]. The motion of the femoral head relative to the acetabular cup is constrained in translational directions due to the contact between the joint surfaces. On the contrary, relative movements in rotational directions are free within the technical RoM. Given these joint characteristics, an appropriate control strategy for the robot is to apply force in the constrained translational and to move the femoral stem in the free rotational directions using hybrid position-force control.

**Fig 1:**
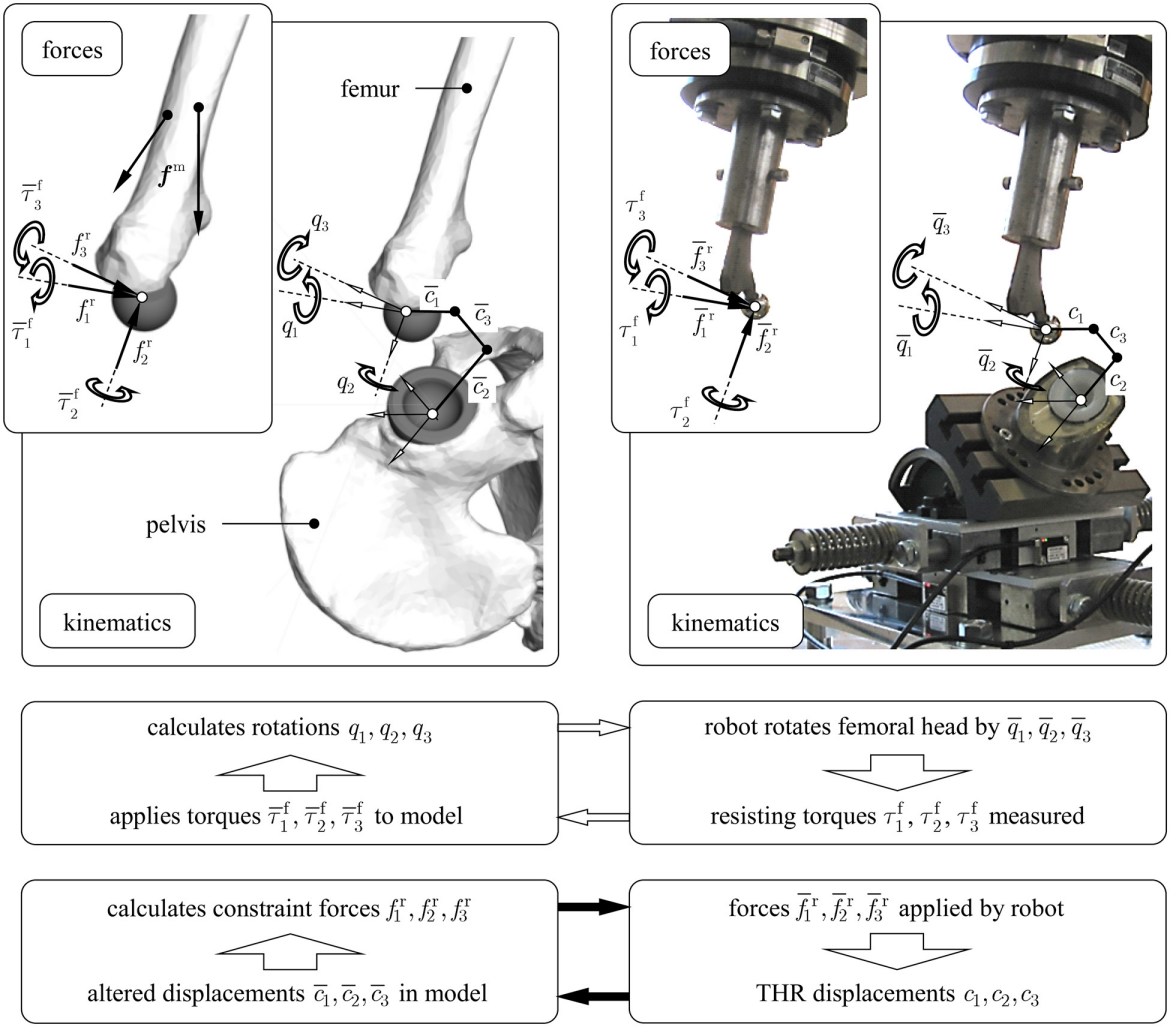
Functional principle of the HiL simulation for testing THR with respect to dislocation. The transfer between the musculoskeletal model and the physical setup is illustrated within the two control loops on kinematic and force level, respectively. The THR components are attached to mounting devices which are fixed to the endeffector of the robot (stem) and the compliant support (cup).

For the spatial load case (Fig 1), the free directions are specified as the rotations of the femur with respect to the pelvis with the angles *q*_1_ (adduction/abduction), *q*_2_ (internal/external rotation) and *q*_3_ (flexion/extension). At a current time instant *t*, the musculoskeletal model delivers values of the angles *q*_1_, *q*_2_ and *q*_3_ which are transferred to the robot controller. Accordingly, the femoral component of the THR is rotated in the position with angles ${\bar{q}}_{1}$, ${\bar{q}}_{2}$ and ${\bar{q}}_{3}$ by the robot under position control. The transferred values denoted by bars normally differ from the original values without bars due to signal delays and the limited dynamic bandwidth of the controlled robot. Resisting torque components $\tau_{1}^{f}$, $\tau_{2}^{f}$, $\tau_{3}^{f}$ usually due to friction, are measured along the coordinates ${\bar{q}}_{1}$, ${\bar{q}}_{2}$ and ${\bar{q}}_{3}$ as a consequence of the movement, and fed back to the model closing the first control loop of the HiL simulation.

The second control loop is given by considering the three translations treated as constrained directions. At the same time instant *t*, the corresponding components of the reaction forces $f_{1}^{r}$, $f_{2}^{r}$, $f_{3}^{r}$ are calculated by the model which mainly depend on active muscle forces ***f*** ^*m*^, gravitational and dynamic forces. After transmission to the robot controller, the robot applies the reaction force components ${\bar{f}}_{1}^{r}$, ${\bar{f}}_{2}^{r}$, ${\bar{f}}_{3}^{r}$ onto the THR using force control. As long as the THR bears the applied forces, only minor relative displacements of the implant components *c*_1_, *c*_2_, *c*_3_ are recorded between the femoral head and the cup. Separation of the joint partners and hence instability is indicated by increasing relative displacements *c*_1_, *c*_2_, *c*_3_. This is also accompanied by rising values of the measured resisting torques $\tau_{1}^{f}$, $\tau_{2}^{f}$, $\tau_{3}^{f}$ in case of impingement [13, 14]. The resisting torques ${\bar{\tau }}_{1}^{f}$, ${\bar{\tau }}_{2}^{f}$, ${\bar{\tau }}_{3}^{f}$ as well as the displacements ${\bar{c}}_{1}$, ${\bar{c}}_{2}$, ${\bar{c}}_{3}$ transferred into the model have an impact on soft tissue elongation and muscle force calculation in the next time instant.

### Physical setup and control

**Fig 2:**
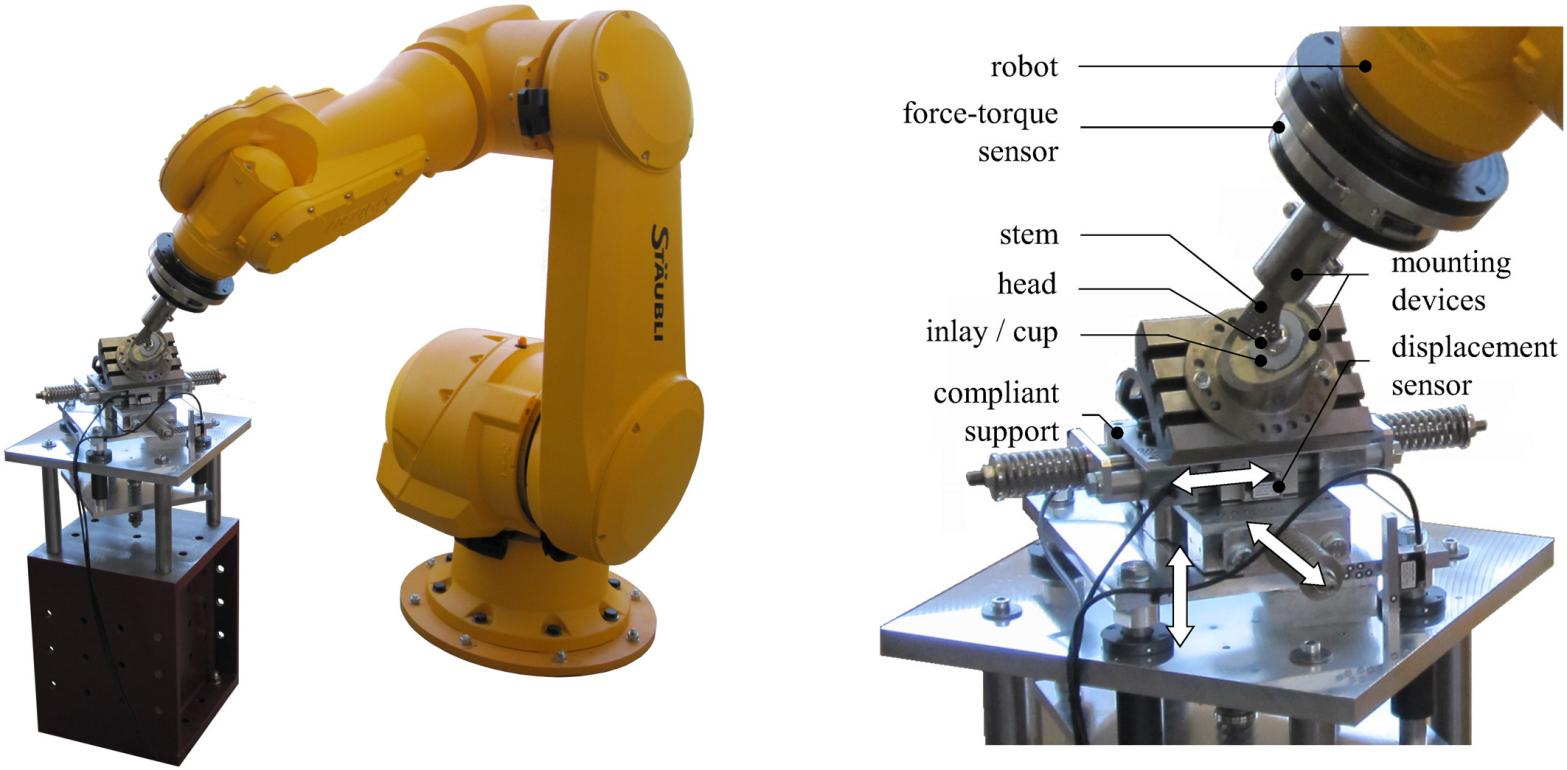
Physical setup of the HiL test system for testing THR. The THR components are fixed on mounting devices attached to the endeffector and the compliant support, respectively. Measurements are taken via the force-torque sensor and displacement sensors.

The physical setup consists of a a six-axis industrial robot (TX200, Stäubli Tec-Systems GmbH, Bayreuth, Germany) equipped with a six degree-of-freedom force-torque sensor (Omega 160, ATI Industrial Automation, Apex, North Carolina, USA) and a compliant support mounted on a ground-fixed framework (Fig 2) [37, 38]. The support consists of three serially arranged prismatic joints with orthogonal axes. Springs restrain the displacements along these axes providing elastic compliance in the three translational directions which are recorded by displacement sensors (MSK 5000, SIKO, Buchenbach, Germany).

Within this work, a standard femoral stem (SL-Plus, size 6, cone 12/14, CCD angle 131°, Smith & Nephew Orthopaedics AG, Baar, Switzerland) with a metallic head (size 28/M) was fixed with polyurethane casting resin into the mounting device attached to the endeffector of the robot. Likewise, an acetabular cup (Alloclassic CSF, size 52, with a polyethylene inlay, size 52/58, Zimmer GmbH, Freiburg, Germany) was mounted on the compliant support. Relative displacements between the femoral head and the cup are obtained by subtracting the data recorded by the displacement sensors from the position of the endeffector indicated by the robot controller.

Under high loads, displacement deviations due to small elastic deformation of the femoral component may occur. These are diminished by an error model implemented into the control program. The error model was calibrated by using a contactless stereo camera system (PONTOS, GOM mbH, Braunschweig, Germany) that measures the spatial positions of the implant components with an accuracy of about 0.01 mm.

To move and load a THR according to its free and constrained directions, the robot runs in hybrid position-force control. Position control is achieved by the robot controller (CS8C HP, Stäubli Tec-Systems GmbH, Bayreuth, Germany) running with a control cycle of 8 ms. Outer force regulating control loops are used for force control generating the control input for the inner position and velocity controllers. This means that the robot moves in the constrained directions until the forces, applied onto the compliant support and measured by the force-torque sensor, coincide with the corresponding desired value [39]. Moreover, torques occurring along the free directions are measured by the force-torque sensor.

During HiL simulations the physical setup and the musculoskeletal model are embedded into a control system [34, 38] which enables the information transfer between all components involved. Within the control system, the robot, the sensors and the model communicate in the same time frame based on the control cycle of the robot controller. The step size of the solver in the model corresponds to the control cycle or a fraction of it. All information are exchanged between model and robot before the beginning of a new time step. The robot receives the desired values from the last computational step of the model and sends current measurements. During computation of the next time step, the robot moves and loads the attached implant components according to the desired values received.

### Musculoskeletal model for THR testing

During a HiL simulation, the musculoskeletal model calculates the reaction forces in the artificial joint for a given human motion based on an inverse dynamics approach. It also has to account for the soft tissue response during an instability event. Hence, capsular, ligament and muscle structures and their respective forces have to be incorporated into the model as well as geometric proportions and inertial properties of the skeletal system.

#### Model topology and coordinates

**Fig 3:**
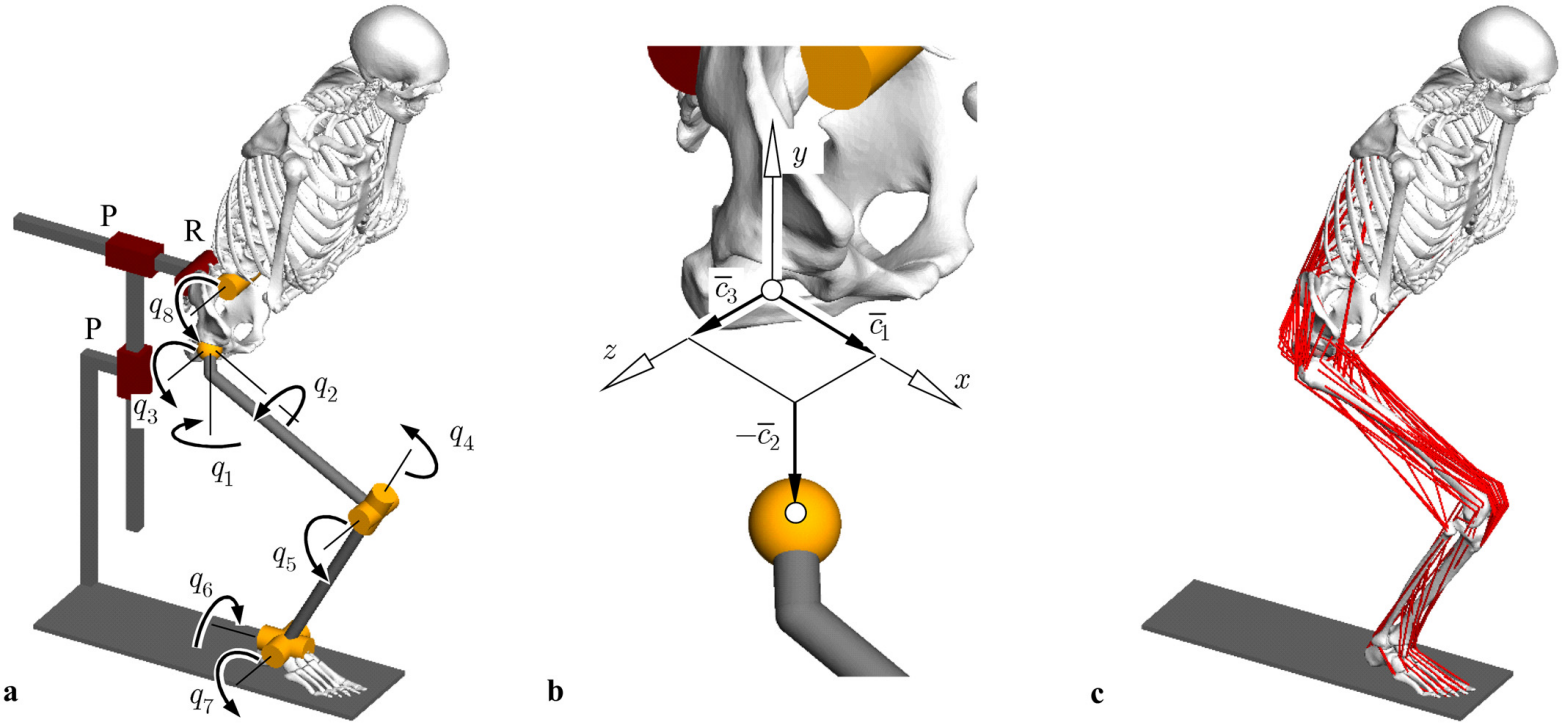
Multibody system of the lower extremity for testing THR. (a) Multibody topology with illustration of the joint coordinates and the fictive planar joint in the sagittal plane indicated as one revolute (R) and two prismatic (P) joints. (b) Measured and transferred coordinates ${\bar{c}}_{1}$, ${\bar{c}}_{2}$, ${\bar{c}}_{3}$ in constrained directions of the THR. (c) Musculoskeletal model with implanted CAD geometries of the THR.

The musculoskeletal model consists of a multibody system with overall four moving segments modeled as rigid bodies (Fig 3a). The kinematic chain starts at the right foot assumed to be ground-fixed and continues with the tibia and fibula summarized as one segment, the femur, the pelvis and the upper body which includes the head, the torso and the upper extremities [40]. Both ankle and knee joint are modeled as universal joints with two rotational degrees of freedom each [41, 42]. The upper body is attached to the pelvis at the sacrum endplate center by a revolute joint. A kinematic subchain represents the hip joint consisting of three orthogonal prismatic joints and three revolute joints with co-intersecting axes [38]. The three coordinates of the prismatic joints are constrained by the measurements $\bar{c}$ which are transferred from the robot to the model (Fig 3b). The three revolute joints correspond to the free rotations defined by the Cardan angles *q*_1_, *q*_2_ and *q*_3_. Moreover, the kinematic chain is closed [43] by means of a fictive planar joint consisting of one revolute (R) and two prismatic (P) joints. It is established in the sagittal plane and connects the pelvis with the ground. The three constraint reactions of the planar joint represent the reactions between the modeled right lower extremity and its left counterpart under the assumption of movements being symmetrical with respect to the sagittal plane with both feet attached to the ground.

The motion of the kinematic chain can be described by the joint coordinates ***q*** = [*q*_1_ … *q*_8_]^T^ and the measured displacements $\bar{c}=[{\bar{c}}_{1}\;\;{\bar{c}}_{2}\;\;{\bar{c}}_{3}]{}^{T}$. The joint coordinates ***q*** are constrained by three implicit loop closure constraints representing the symmetry conditions with respect to the sagittal plane. The constraints read at the position and velocity levels, respectively,

$$g(q,\bar{c})=0\;,$$ (1)

$$\dot{g}\equiv G\left(q,\bar{c}\right)\;\dot{q}=0\;\text{with}\;G=\frac{\partial g}{\partial q}.$$ (2)

The constraints Eq (1) also depend on the measured displacements $\bar{c}$ in the constrained directions of the hip joint, see Fig 1. In Eq (2) their time derivative $\dot{\bar{c}}$ is neglected as the dynamics of the measured displacements $\bar{c}$ during the HiL simulation is not physically based but governed by the force controller of the robot. This assumption is considered to be acceptable as long as the displacements $\bar{c}$ are small.

#### Equations of motion

The equations of motion of the model are formulated in terms of the joint coordinates ***q*** and the measured displacements $\bar{c}$,

$$M\left(q,\bar{c}\right)\;\ddot{q}=\tau^{c}\left(q,\dot{q},\bar{c}\right)+\tau^{p}\left(q,\dot{q},\bar{c}\right)+{\bar{\tau }}^{f}+\tau^{m}\left(q,\bar{c},\alpha \right)+\tau^{r}.$$ (3)

The (8, 8) mass matrix ***M*** is obtained under the assumption that the soft tissue masses are added to the masses of the corresponding skeletal segments. Dynamic wobbling of the muscle masses is neglected. The vector ***τ*** ^c^ contains the torques of the centrifugal, gravity and contact forces with respect to the joint axes, ***τ*** ^p^ are the joint torques due to passive soft tissue structures such as the hip capsule, ${\bar{\tau }}^{f}$ are the measured resisting torques fed back from the robot into the model according to Fig (1), and ***τ*** ^m^ are the joint torques from the active muscle forces depending on muscle activation levels ***α***. The vector ***τ*** ^r^ includes the joint torques due to the constraint forces at the cut planar joint that are expressed by means of the (3, 8) Jacobian matrix ***G*** from Eq (2) and the vector ***λ*** = [*λ*_1_ *λ*_2_ *λ*_3_]^T^ with the constraint force coordinates (Lagrange multipliers) of the planar joint,

$$\tau^{r}=G{}^{T}\left(q,\bar{c}\right)\;\lambda .$$ (4)

#### Inverse dynamics

The equations of motion Eq (3) are used to calculate the muscle forces and the hip joint reaction force for given motion ***q***(*t*) according to an inverse dynamics approach. Here, two sources of redundancy occur in the musculoskeletal system. The first redundancy is due to the closed kinematic chain of the model representing human motions with both feet attached to the ground [43]. This means physically that the musculoskeletal system can be internally loaded by active muscle forces without generating motion. The second redundancy is due to the distribution problem of muscle forces as generally more muscle structures are available than required to produce muscle torques for a specific motor task [44, 45].

For a given motion ***q***(*t*) and its time derivatives satisfying the loop closure constraints Eq (1) and their time derivatives, the first redundancy problem is addressed by evaluating the equations of motion Eq (3) with respect to the eight muscle torques ***τ*** ^*m*^ and the three constraint force coordinates ***λ***. The actual displacements in the constrained directions $\bar{c}$ are known from the measurements at the physical setup. This leads to an under-determined linear system of eight equations in altogether eleven unknowns ***τ*** ^*m*^ and ***λ***,

$$\left[\begin{array}{cc} I & G{}^{T} \end{array}\right]\;\left[\begin{array}{c} \tau^{m}\\ \lambda \end{array}\right]=b\;\text{with}\;\;b=M\;\ddot{q}-\tau^{c}-\tau^{p}-{\bar{\tau }}^{f}\;$$ (5)

and the identity matrix ***I***. The redundancy problem is solved by regarding Eq (5) as an equality constraint of a static optimisation problem minimising a quadratic objective function with a diagonal weighting matrix ***Q*** [46],

$$Z\left(x\right)\equiv x{}^{T}Q\;x=\min_{x}\;\text{with}\;\;x=\left[\begin{array}{c} \tau^{m}\\ \lambda \end{array}\right]\;\text{subjected}\;\text{to}\;(5).$$ (6)

The second redundancy problem has to be solved for estimating *n* individual muscle forces from the muscle torques ***τ*** ^*m*^. To incorporate muscle architecture, the forces of *n* muscle force elements ***f*** ^m^ = [*f*_1_ … *f_n_*]^T^ are expressed in terms of their corresponding muscle activation levels ***α*** = [*α*_1_…*α*_*n*_]^T^ which are used to scale the isometric force of each muscle [40]. Hence,

$$f^{m}=B\left(q,\bar{c}\right)\;C\;\alpha \;.$$ (7)

Here, the (*n*, *n*) matrix ***B*** summarizes the normalized force directions of each muscle. Matrix ***C*** = diag(*C*_1_ … *C*_*n*_)contains the isometric muscle forces obtained from the physiological muscle cross section area *A*_*i*_ times the physiological muscle stress *σ*_*i*_,

$$C_{i}=A_{i}\;\sigma_{i},\;i=1,\ldots ,n\;.$$ (8)

With the (1, 8) Jacobian matrices $J_{i}^{m}$, which relates the length rate of the *i*th muscle ${\dot{s}}_{i}$ with the time derivatives of the joint angles $\dot{q}$, thus ${\dot{s}}_{i}=J_{i}^{m}\;\dot{q}$, the contribution of the *i*th muscle force $f_{i}^{m}$ to the muscle torques is described by $\tau_{i}^{\text{m}}=J_{i}^{m\;\text{T}}\;f_{i}^{\text{m}}$. Together with Eq (7), the distribution problem is then formulated as an under-determined system of eight linear equations for *n* muscle activation levels ***α***,

$$J^{m\;T}\left(q,\bar{c}\right)\;B\left(q,\bar{c}\right)\;C\;\alpha =\tau^{m}\;\text{with}\;J^{m}=\left[\begin{array}{c} J_{1}^{m}\\ \vdots \\ J_{n}^{m} \end{array}\right]$$ (9)

with muscle torques ***τ*** ^m^ obtained from Eq (6). Again a static optimization problem is solved. Here, a quadratic cost function with a positive definite weighting matrix ***P*** is considered,

$$Z_{\alpha }\left(x\right)\equiv \alpha {}^{T}P\;\alpha =\min_{\alpha }\;\text{with}\;\;\alpha \;\text{subjected}\;\text{to}\;(9)\;$$ (10)

and 0 ≤ *α*_*i*_ ≤ 1 [40].

Finally, the reaction force ***f*** ^r^ at the hip joint is calculated using the laws of momentum and moment of momentum of the bodies of the kinematic chain with the reaction force coordinates obtained from Eq (6) and the muscle forces from Eq (7).

#### Implementation of the musculoskeletal model

Bone geometries were derived from a human male computed tomography dataset [47] using segmentation and reconstructing techniques [48] (Fig 3c). Reconstructed geometries were transformed into local reference frames [49–51]. Joint rotation centres were obtained by fitting spheres or cylinders into articulating surfaces of the geometries. Subsequently, the kinematic chain described above was composed in the multibody software SIMPACK (Version 8.9, Simpack AG, Gilching, Germany). The overall segment masses *m*_*i*_ were formulated as functions of the subject’s mass using regression equations [52] which were further differentiated into bone and soft tissue masses.

As implantation of THR components may alter the geometric proportions within the kinematic chain, CAD geometries of the acetabular cup and the femoral stem were virtually implanted into the bone structures introducing implant positioning and design parameters. The cup was placed according to radiographic angles (cup inclination *ι*, cup anteversion *β*) with respect to the pelvic reference frame [9, 53] preserving the hip joint rotation center on the pelvic side. The stem was positioned along the long axis of the proximal femur whereas the neck-shaft intersections of both implant and bone served as reference points for implant setting *s*. Stem antetorsion *ϑ* was defined as rotation around the implant shaft axis aligned to the long axis of the proximal femur shaft. At *ϑ* = 0° anteversion, the implant neck axis fell into a plane spanned by the most posterior points of the two condyles and the greater trochanter. For given design parameters (CCD angle *ν*, neck length *l*, head offset *h* and head diameter *d*), the hip joint rotation center was defined with respect to the femoral reference frame which does not necessarily coincide with the center of the native femoral head.

Passive forces from capsule and ligament structures were neglected, i.e. ***τ*** ^p^ = **0**. Active muscle forces were assumed to act along straight lines [45]. Hence, overall *n* = 70 muscle elements were implemented according to anatomic attachment sites [54] whereas larger muscles were split into several elements. To avoid bone intrusion and intersection of joint rotation centers even for higher degrees of flexion, muscle wrapping and curvature were taken into account by using segment-fixed via-points [55]. Likewise, deflection of the quadriceps apparatus was modeled by femur-fixed via-points along the trochlear groove gained from a patella-femoral model [56]. Physiological cross section areas *A*_*j*_ were derived from the literature [57, 58]. Physiological muscle stresses were assumed to be *σ*_*i*_ = 1.0 MPa [59] for all muscle force elements. Smooth contact [60] was implemented between the global reference frame and the tuber ischiadicum depending on seat height and body weight. Moreover, a quadratic programming algorithm [61] was implemented into the model to resolve both redundancy problems given in Eqs (6) and (10), respectively.

### Motion analyses and model parametrization

As in vivo motion data of dislocation-associated leg maneuvers were not available, motion analyses with one healthy human subject (male, 24 a, 1.81 m, 80 kg) were performed. Written informed consent was obtained from the subject. The study and the consent procedure were approved by the Local Ethics Committee of the University of Rostock (A 2010–84). The investigations were conducted according to the principles of the Declaration of Helsinki.

During the analyses, kinematic data of skin markers placed on palpable bony landmarks [62] were recorded using an ultrasound measuring system (CMS-HS Measuring System, zebris Medical GmbH, Isny im Allgäu, Germany). Two leg maneuvers were considered:

1. normal sitting down and standing up (48 cm seat height) for validation purposes
2. deep sitting down and standing up with femoral adduction (33 cm seat height) for analyzing dislocation behavior

The deep maneuver is associated with high risk of anterior impingement and posterior dislocation [18]. Both maneuvers were repeated five times. Averaged data sets were gained for each maneuver by normalizing the time scale and averaging the five motion cycles. Down sampling, spline interpolation and numerical differentiation were performed using the Curve Fitting Toolbox in MATLAB (Version 7.11, MathWorks, Ismaning, Germany) to achieve smooth kinematic data of the skin markers from the averaged data sets at the position, velocity and acceleration levels. MRI data of the lower extremities were recorded along with the attached skin markers after the motion analyses.

Subsequently, the musculoskeletal model was scaled onto the subject from the motion analyses according to the osseous anatomic structure derived from the MRI data recorded and body weight. Likewise, segment-fixed points were generated in the model based on the position of the skin markers relative to the bones. The model was then parametrized with varying maneuvers, implant positions (inclination *ι*, cup anteversion *β*, stem antetorsion *ϑ*), subject’s body mass, and muscle elements *n*, resulting into several model variations (Table 1). Design parameters (CCD angle *ν* = 131°, neck length *l* = 53.9 mm, head offset *h* = 0 mm, head diameter *d* = 28 mm) and implant setting (*s* = −5 mm) were kept constant. For each variation the corresponding measured maneuver was transferred onto the joint coordinates of the model ***q*** by means of a model-based least squares fit. The distances between skin markers and related segment-fixed points were minimized by coupled springs [37], where the cost function could be interpreted as potential of virtual springs between trajectories of the skin markers and compliant trajectories of the segment-fixed points. This procedure is equivalent to global optimization techniques which were shown to reduce skin motion artifacts [63, 64]. Hence, a consistent set of joint coordinates ***q***(*t*) was obtained for each variation from the averaged and smoothed kinematic data.

**Table 1:** Configurations for nine HiL simulations with varying model and test parameters.

no.	maneuver	lubrication	cup inclination *ι*	cup anteversion *β*	stem antetorsion *ϑ*	body mass	muscle elements *n*
①	normal	dry	45°	0°	25°	100%	70
②	deep	dry	60°	20°	−10°	100%	70
③	deep	water	60°	20°	−10°	100%	70
④	deep	water	45°	20°	10°	100%	70
⑤	deep	water	60°	0°	−10°	100%	70
⑥	deep	water	45°	0°	−10°	100%	70
⑦	deep	water	45°	0°	−10°	75%	70
⑧	deep	water	45°	0°	−10°	50%	70
⑨	deep	water	60°	0°	−10°	100%	62

### Configurations and validation

Overall, nine HiL simulations were configured with varying model and test parameters (Table 1): Normal sitting down and standing up was simulated with parameter set ① for validation of the HiL test system. Based on parameter sets ② and ③, experiments with dry articulating surfaces and with lubrication by deionized water were conducted, to evaluate the influence of friction for the metal-to-polyethylene bearing. For the same deep maneuver, the influence of the implant position (inclination *ι*, cup anteversion *β*, stem antetorsion *ϑ*) on the impingement and dislocation behavior was considered within configurations ③, ④, ⑤ and ⑥. With regard to specific maneuvers to be evaluated in the near future, it might occur that the calculated loads exceed the calibrated measurement range of the force-torque sensor. Therefore, scaling of the subject’s body mass may become necessary, as addressed in parameter sets ⑥, ⑦, ⑧. Furthermore, the impact of removing muscle structures on the dislocation process was exemplified in parameter set ⑨. All small external rotators of the hip joint (Mm. gemelli, Mm. obturatorii, M. piriformis and M. quadratus femoris) were removed from the model emulating resection of these muscles as performed during a posterior surgical approach [65].

All model variations were exported into real-time capable machine code calculated with 1 ms as fixed time step size. A scaling factor was introduced between simulation time and real time as a control cycle of 8 ms was used for the robot controller. The exported model variations were embedded into the control system to allow interactions with the physical setup. Before each HiL simulation, the force-torque sensor was calibrated for the given joint motion by subtracting the resulting torque due the endeffector’s weight at the femoral head. Both initial angular position and force was applied by the robot according to the initial values of the model. At this position, all values of the measured relative displacements *c* were reset to zero. Finally, the HiL simulation was initialized by the model. To assure reproducibility, each HiL simulation was repeated three times.

For validation purposes, hip contact forces were derived from in vivo measurements of routine activities [33]. These included trials from three male patients with instrumented THR for sitting down and standing up activities (chair height 50 cm). The force data were transformed to a standardized global reference frame [66] and normalized with respect to time. As the patients exhibited varying timing, the force components and resultants were shifted and rearranged for each trial with respect to the maxima of their resultant used as reference point [67]. Mean values and standard deviations (±SD) were estimated for each patient and activity, which enabled a subject-to-patients comparison [68] with the outcomes of the HiL test system configured with parameter set ①. Furthermore, two validation requirement were defined: First, reproduction of major trends for all force components given by the envelopes of the in vivo data of the three patients; and second, prediction of force levels comparable to the in vivo hip contact forces.

## Results

### Validation of the HiL test system

**Fig 4:**
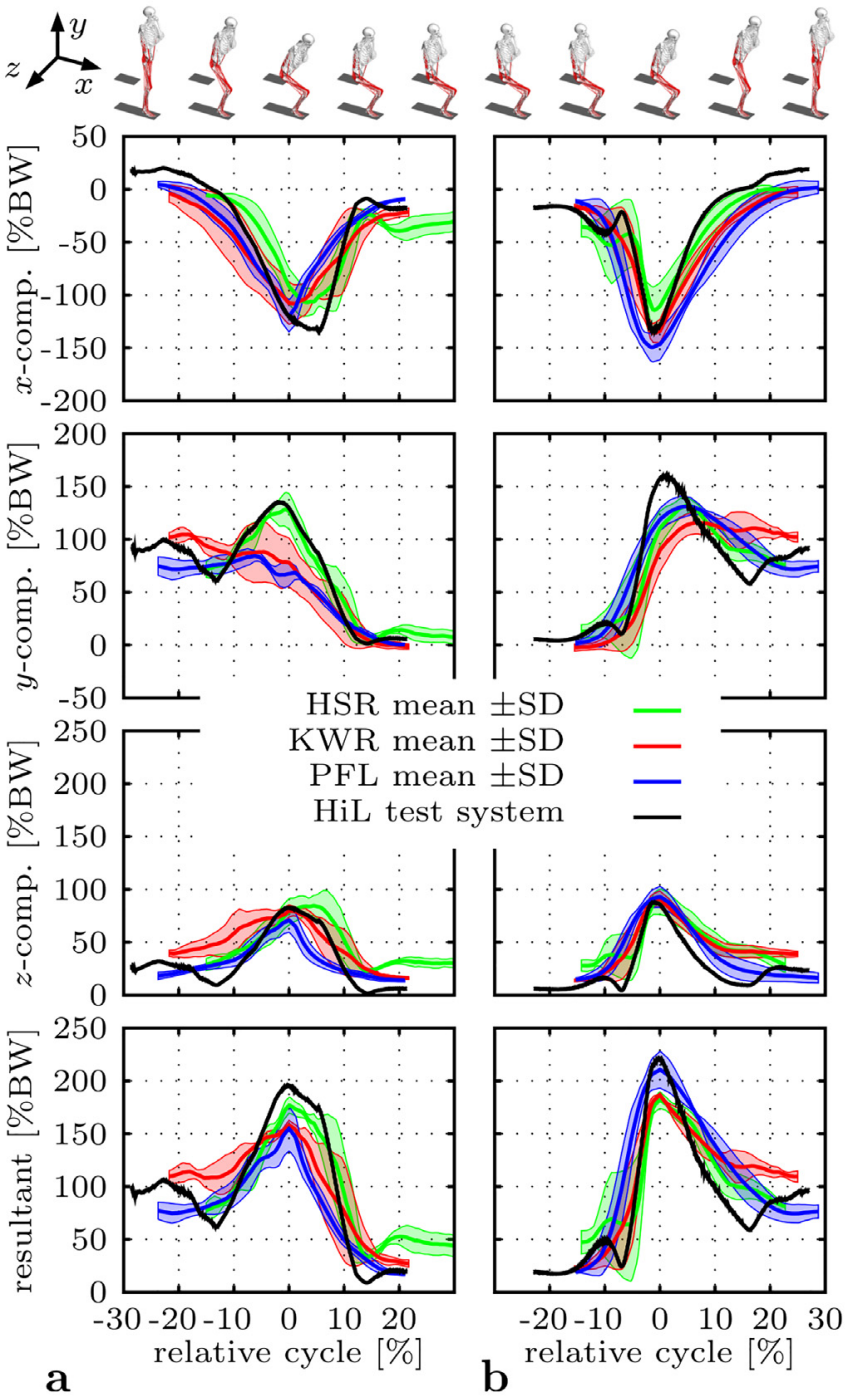
Predicted hip joint reaction force *f* ^*r*^ for the normal maneuver compared to in vivo measurements of three male patients (HSR, KWR and PFL) [33]. All force components are given with respect to the global reference frame [66], shifted and rearranged with respect to the maxima of the corresponding resultant used as reference point [67], and mirrored to the left hip joint [33]. **a** Sitting down. **b** Standing up.

Predicted hip joint reaction forces of parameter set ① (Table 1) were compared to in vivo hip contact forces (mean values ±SD) derived from the three instrumented patients [33] for normal sitting down and standing up activities (Fig 4). Qualitatively, normal sitting down is characterized by a sudden rise in the hip contact force during lowering from the standing position. With increasing chair contact, the force level drops to a plateau reached during actual sitting. The load profile described is reversed in the standing up activity. These force characteristics were present in all force components and resultants of the in vivo measurements, and were reproduced well by the HiL test system. The predicted values fell predominantly into the envelopes spanned by the patients’ data. Differences were noted for the sitting down activity at peak values of the x-component and the resultant, and the z-component during the standing and sitting phase, respectively. Standing up showed discrepancies at peak values of the y-component.

Quantitatively, the HiL test system estimated peak force values of -134.2%BW, 135.0%BW, 82.7%BW and 195.4%BW for x-, y-, z-components and absolute value during sitting down. In comparison to the patients’ measurements, largest deviations for these peak values remained in x-direction ranging from 12.0% to 25.6% (Table 2). The closest match was found with patient HSR differing with an absolute peak value of 10.5% (25.6%, 5.3%, -2.3% in x-, y-, z-direction). Standing up revealed higher force levels reaching 220.8%BW for the peak resultant (-133.7%BW, 160.0%BW, 87.6%BW for x-, y-, z-components). Largest deviations were observed in y-direction overestimating the in vivo data by 21.1% to 38.0% at peak values. According to the peak resultant, the predictions came closest to patient PFL with a deviation of 4.9% (-10.6%, 21.8%, -5.7% for x-, y-, z-components).

**Table 2:** Relative deviations estimated at peak values between predictions of the HiL test system and hip contact forces derived from three instrumented patients [33] for normal sitting down and standing up.

patient	activity	relative deviations [%]			
		x-component	y-component	z-component	resultant
PFL	sitting down	12.0	60.3	17.0	26.2
	standing up	−10.6	21.8	−5.7	4.9
KWR	sitting down	24.1	29.0	3.9	23.7
	standing up	−1.7	38.0	−3.3	18.2
HSR	sitting down	25.6	5.3	−2.3	10.5
	standing up	17.0	21.1	−0.7	21.5

### Impact of joint lubrication

**Fig 5:**
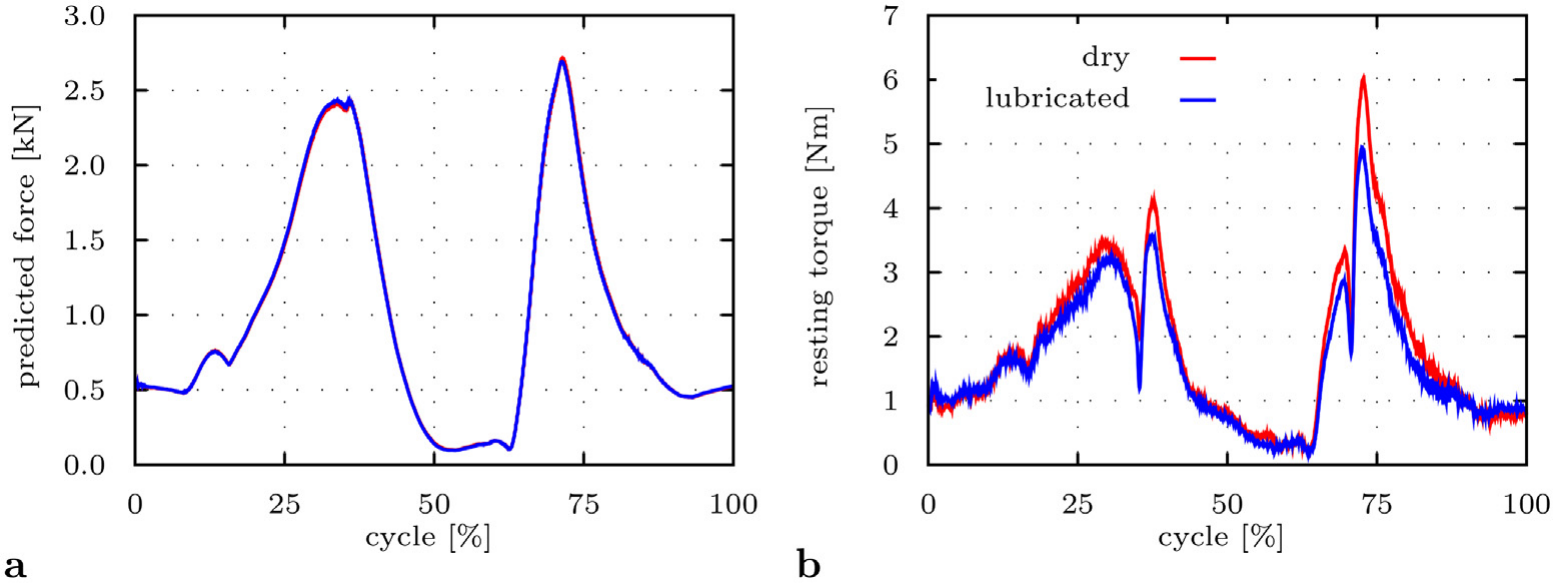
Impact of friction under dry and lubricated conditions on HiL-simulated THR load situation for a deep seating-to-rising motion cycle. The HiL simulations are based on parameter sets ②, ③ from Table 1. **a** Absolute value of predicted reaction force |***f*** ^*r*^|. **b** Absolute value of measured resisting torque |***τ*** ^*f*^|.

For both friction conditions (parameter sets ② and ③), the absolute value of the resisting torque (Fig 5b) was roughly proportional to the absolute value of the joint force (Fig 5a). The brief sharp drops of the resisting torque were caused by changes of the direction of the flexion angle under high load during seating-to-rising. Joint lubrication reduced the absolute value of friction torque over the whole motion cycle (Fig 5b).

### Impact of implant position

**Fig 6:**
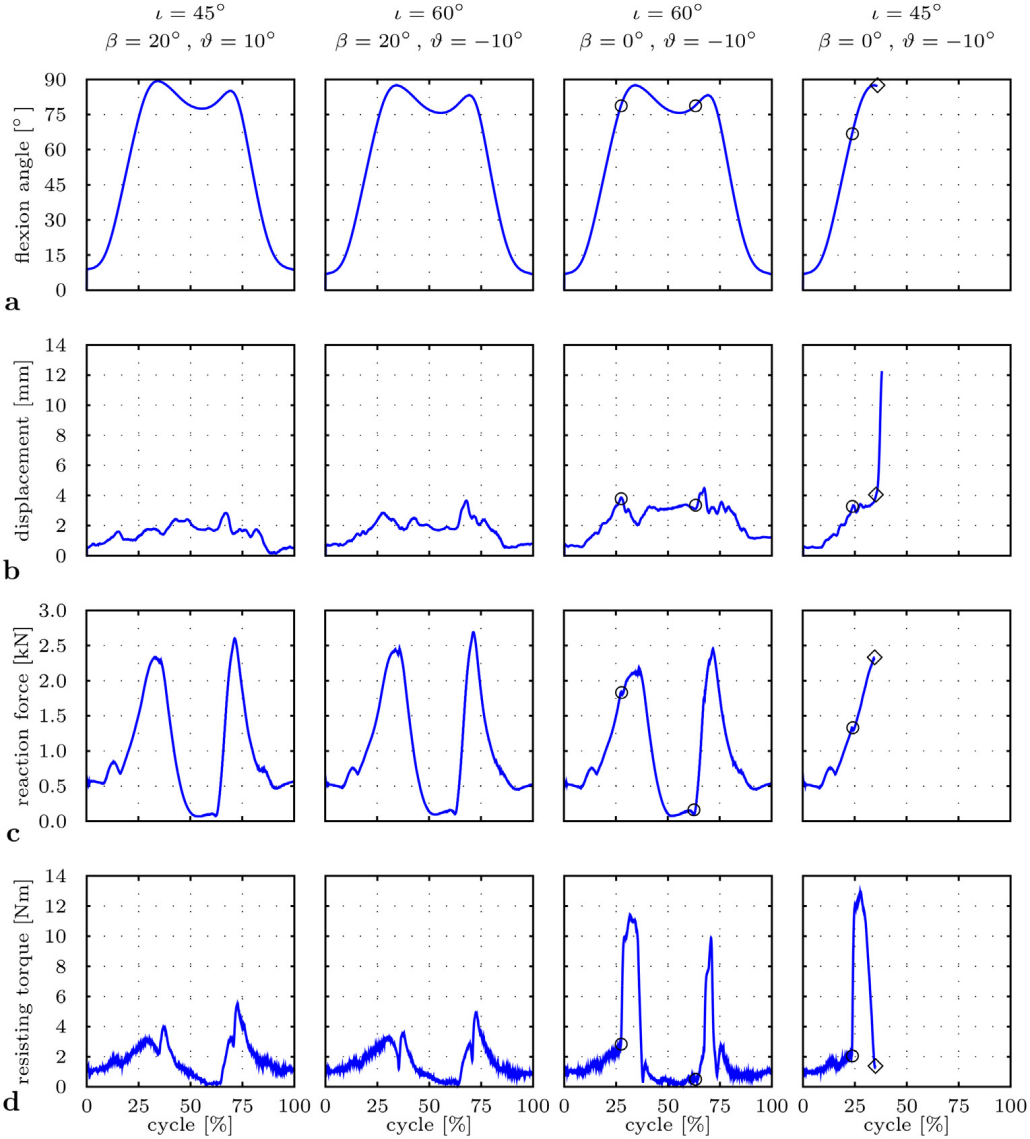
Impact of implant position on HiL-simulated THR load situation for a deep seating-to-rising motion cycle. Implant positions are defined by inclination *ι*, cup anteversion *β*, and stem antetorsion *ϑ* with parameter sets ③, ④, ⑤, ⑥ from Table 1. Impingement occurs at ○ and dislocation at ◇. **a** Flexion angle *q*_3_. **b** Measured displacement |***c***| between femoral head and acetabular cup. **c** Predicted reaction force |***f*** ^*r*^|. **d** Measured resisting torque |***τ*** ^*f*^|.

The flexion angle over time was defined between the femur axis and the frontal pelvis plane, whereby pelvic tilt was taken into account (Fig 6a). Impingements were typically indicated by an abrupt rise of the resisting torque (Fig 6d), caused by the eccentric contact force between the femoral neck and the cup rim. Dislocation became evident by an increasing displacement between the rotation centers of prosthetic head and acetabular cup (Fig 6c).

For parameter sets ③ and ④, each with cup anteversion 20°, neither impingement nor dislocation occurred for cup inclination angles of 45° and 60°. Besides that, alteration of stem antetorsion *ϑ* lead to changed joint angles and hence reaction forces |***f*** ^*r*^| and resisting torques |***τ*** ^*f*^|. Comparing parameter sets ③ vs. ④, it could be shown that 20° less cup anteversion *β* of the cup caused anterior impingement during deep sitting at 78° flexion (27% of the motion cycle). A similar effect occurred, if cup inclination *ι* was decreased, comparing ⑤ vs. ⑥. In this case, 15° less cup inclination *ι* caused 12° decreased degree of flexion until impingement. Comparing parameter sets ⑤ vs. ⑥, the RoM until impingement was reduced both by 20° less cup anteversion *β* and 20° less stem antetorsion *ϑ* (from 10° to −10°), leading from an impingement-free maneuver to anterior impingement and posterior dislocation at 66° and 87° flexion, respectively.

### Impact of subject’s body mass

**Fig 7:**
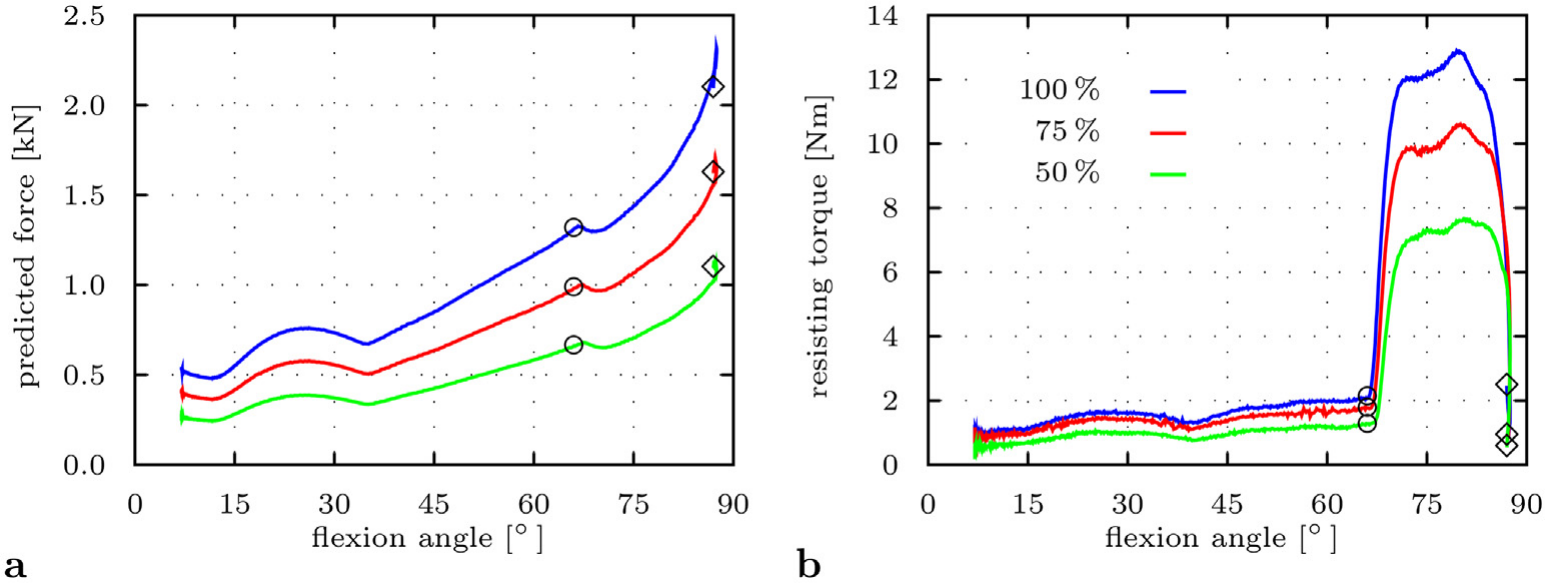
Impact of load level adjusted by body mass on HiL-simulated THR load situation for deep seating-to-rising. The HiL simulations are based on parameter sets ②, ③, ④ from Table 1. Impingement occurs at ○ and dislocation at ◇. **a** Predicted reaction force |***f*** ^*r*^| over flexion angle. **b** Measured resisting torque |***τ*** ^*f*^| over flexion angle.

Reduction of body mass was accompanied with consistent lower amounts of reaction forces and resisting torques over the course of the maneuver (Fig 7). No alterations of RoM until impingement and dislocation were indicated.

### Impact of removing muscle structures

**Fig 8:**
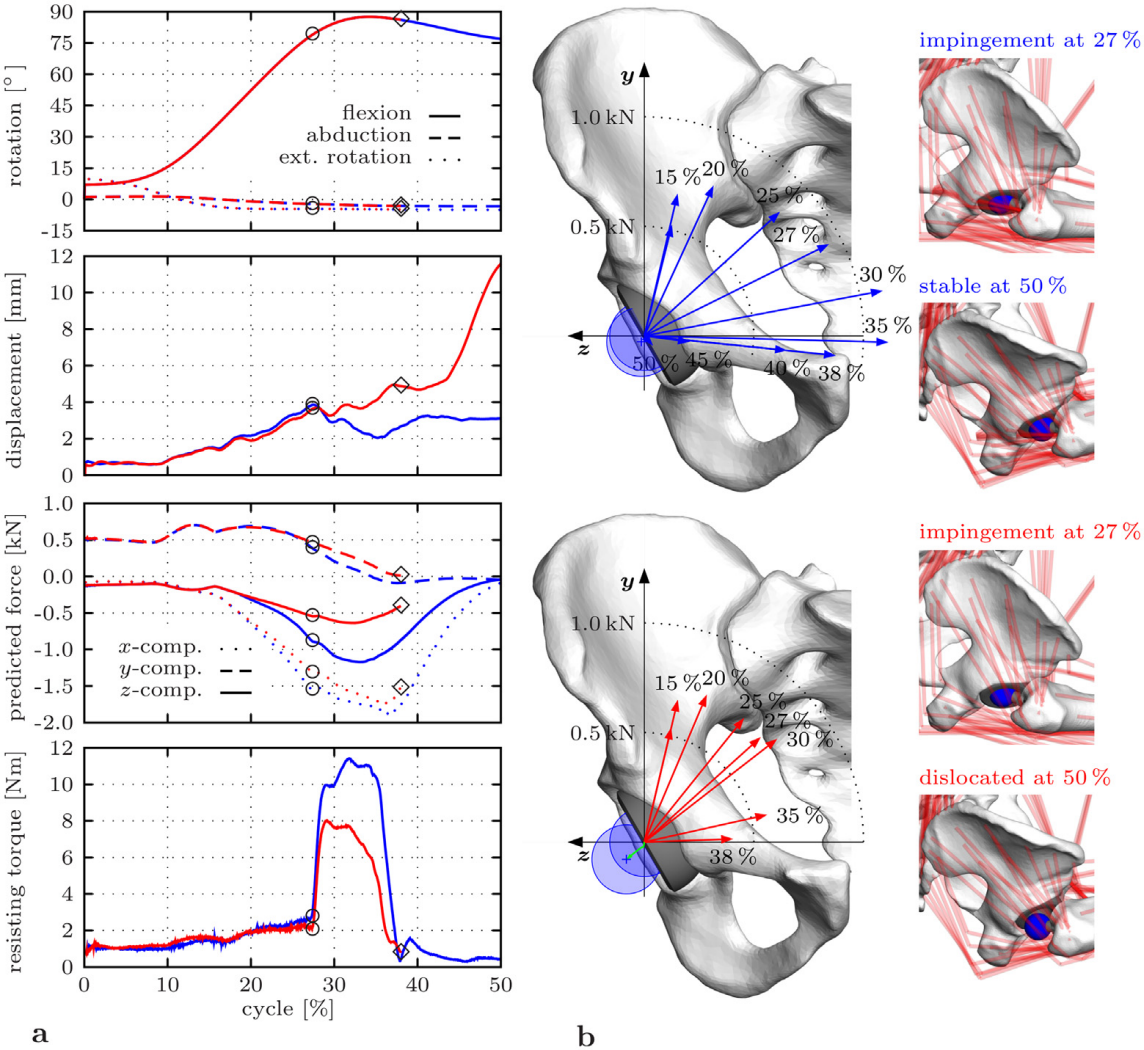
Impact of muscle element removal emulating a posterior surgical approach on HiL-simulated THR load situation with focus on the sitting down phase of the deep maneuver. The HiL simulations are based on parameter sets ②, ③ from Table 1. **a** Comparison between the intact (blue lines) and the resected (red lines) case for hip joint rotations *q*_3_, *q*_1_, *q*_2_, measured displacement |***c***| between femoral head and acetabular cup, components of the predicted reaction force ***f*** ^*r*^ given in the pelvic reference frame [49], and measured resisting torque |***τ*** ^*f*^|. Impingement occurs at ○ and dislocation at ◇. **b** Direction of the hip joint reaction force with respect to the frontal plane of the pelvic reference frame [49] with illustration of the head position at and after impingement for the intact (above) and the resected (below) case.

The outcomes of the emulated posterior surgical approach were contrasted with the results of the HiL simulation with the same implant position, but intact muscles (parameter set ③ vs. ④). The rotational motion remained identical between the two variations throughout the considered maneuver (Fig 8a). Both indicated impingement after 78° hip flexion (27% of the motion cycle). For the posterior approach, the femoral head dislocated after 85° hip flexion (38% of the motion cycle), in contrast to the intact case where the head remained in the cup. The dislocation process observed was accompanied by a lower load level of the reaction force and the resisting torque. The reduced load level was mainly due to the mediolateral force component dropping at peak values from −1172 N for the intact to −638 N for the resected case. The alterations within the force components was reflected by changes in force directions (Fig 8b).

## Discussion

In the present work, a novel HiL simulation approach was used to analyze the impact of certain test conditions on a potential THR instability maneuver. Within a selected set of parameters, these test conditions included variation in joint lubrication, implant position, subject’s body mass and muscle structures. Lubrication of the artificial joint was shown to cause less friction torques. This is supported by data of Bishop et al. [69] who examined friction characteristics of hard-hard bearings. As regards implant position, it could be demonstrated that less cup anteversion and inclination lead to earlier impingement in flexion motion, as confirmed by previous studies [4, 8]; even though these had not considered realistic musculoskeletal conditions as well as pelvic tilt. Apart from the load level, no influence of body mass was found on the impingement and dislocation behavior in the analyzed parameter sets. This means that downscaling of subject’s body mass may be acceptable for further studies with regard to the limited calibrated measurement range of the force-torque sensor. Moreover, emulation of a posterior surgical approach indicated alterations in THR loading and the instability process in contrast to a reference case with intact muscles. Van Arkel et al. [70] revealed that most of the external hip rotators cannot contribute to edge loading solely based on their lines of action. This may explain the lower load level observed especially in mediolateral direction when removing the small external rotators, which directed the hip joint reaction force more towards the entry plain of the cup. Hence, a reduced force closure was achieved between the THR components than with intact musculature, which seemed to promote levering out of the femoral head after impingement for the considered implant position and maneuver.

One of the key issues for considering realistic THR dynamics is, how to adequately address muscle forces such that anatomic and physiological conditions are met in a testing or simulation environment. Whereas clinical studies are primarily limited as many crucial factors cannot be maintained on a constant and independent level, testing of real components or simulations using mathematical models are suitable approaches for illuminating cause and effect on a systematic basis. But both also entail certain short-comings.

Actuated along one or more directions, mechanical setups allowed for application of constant [13, 71] or prescribed load patterns [11, 12] onto THR components. It remains an open question how passive and active soft tissue structures can be adequately accounted for based on these setups. In contrast to that, specimen-based testing promises incorporation of real soft tissue such as ligaments and capsular structures, including their mechanical response [30–32]. Muscle forces were often emulated statically by cable and pulley systems [15, 16]. However, the muscle forces applied may not reproduce in vivo conditions, not to mention the consideration of all relevant muscle structures spanning the hip joint. Moreover, human specimens do not permit reproducible and comparable evaluation of various parameters under exactly the same boundary conditions due to their decay after extraction.

In addition to testing procedures, mathematical models have been established to be useful tools for biomechanical investigations. Once deployed, simulations can be almost infinitely repeated and parameters arbitrarily varied. Several analytic approaches [5–9, 72] assisted to study joint kinematics with respect to their technical RoM without consideration of soft tissue structures. Other studies [4, 14, 15, 19, 32] focused on stresses and strains acting in THR components during the dislocation process by using finite element analysis. Yet, models including contact mechanics are afflicted with persistent uncertainties concerning the contact conditions.

More comprehensive approaches as regards physiological conditions relied on musculoskeletal models. Higa et al. [17] detected dislocation for passive motions by impingement or an outwardly directed vector of the joint reaction force from the cup’s entry surface. Besides the lack of active muscle forces, there is unfortunately no way to know whether a given impingement event or traction force will or will not presage dislocation based on their approach. On the contrary, Nadzadi et al. [18] obtained hip joint kinematics of several subjects and corresponding force data derived from a validated musculoskeletal model [73]. The data served as boundary conditions for several finite element analyses which aimed to evaluate dislocation risk for realistic maneuvers of the lower extremity [18, 19, 21, 22, 32, 74, 75].

This approach, however, bears a major shortcoming, apart from common limitations arising from finite element modeling. Prescribed motion as well as loading used as boundary conditions imply the assumption that femoral head and acetabular cup remain concentrically aligned throughout the entire simulation of a dislocation-associated maneuver [18]. This means that no interdependency between motion and forces is taken into account during events of instability which may potentially change the subluxation processes investigated. Consequently, it remains unclear whether rising resisting torques or soft tissue forces can actually prevent the prosthetic head from dislocating, which especially applies for spontaneous separation. Elkins et al. [22, 75] even neglected that alterations in femoral parameters such as neck length and stem antetorsion entail changes in overall musculoskeletal dynamics and hence the load situation at the hip joint as outlined by several researchers [23–26].

In this sense, reproduction of THR dynamics while accounting for muscle activity and passive soft tissue response may remain an irreconcilable challenge based on the outlined approaches. It seems impossible to comply with these demands within an experimental test setup alone. Mathematical models may approximate in vivo conditions to a valid extent, though complex contact modeling is limited. These trade-offs led to consideration of alternative approaches which resulted into the HiL test system presented.

Several limitations need to be mentioned with respect to the HiL test system in the current state. First, integration of force control along constrained directions of THR means that the dynamics of the respective movements are not governed by the equations of motion of the embedded multibody model. Instead, these are based on the settings of the force controller [36]. This assumption is considered to be acceptable for minor displacements/rotations compared to the motion in the free directions. That is especially the case as long as the robot is able to apply the desired force values onto the THR components, for instance during subluxation.

Second, the use of a robotic actuator system within HiL control loops implies further demands on the testing procedure. Krenn and Schäfer [76] evaluated the stability of HiL simulations using an industrial robot to emulate the dynamic behavior of a manipulator during contact operations with a real object in space. After varying influencing parameters within a theoretical example, they concluded that large delay times and low sampling rate may cause instabilities of the HiL simulation. In a similar context, Boge and Ma [77] specified two conditions required for high fidelity of contact maneuvers within a robot-based HiL simulation: fast response of the robot to the control command, and same dynamic behavior of the robot’s endeffector and the embedded multibody model during contact. The time between command and execution is given by the robot’s responding time which was between four to eight times the control cycles in their study. Boge and Ma [77] also stated that industrial robots would not completely conform to the second condition for their HiL application due to their high stiffness.

As all issues concerning delay times, sampling rate and potential instability of the HiL control loops specifically depend on the industrial robot and its control system, the functionality of the HiL test system was verified in previous studies. Kähler et al. [36] tested both position and force control modes by embedding a simulated spring-damper oscillator with one degree of freedom into the HiL environment. Concerning THR stability, functionality of the hybrid position-force control was assured by simulating the dislocation process of a standard THR under prescribed boundary conditions [34]. The outcomes of this study showed overall superior performance compared to results of a mechanical setup [13] in terms of measuring sensitivity and reproducibility. Using the same operation mode, Fabry et al. [11] reproduced physiological loading conditions to evaluate the dynamics of tripolar THR systems. Moreover, the functional principle of the HiL approach was proven for testing THR stability with respect to a deep squatting maneuver [37].

Third, musculoskeletal modeling rests upon a rigid multibody approach which involves idealizations and simplifications of the real biomechanical system. Emphasis was placed on well reflecting the geometric proportions and degrees of freedom of the real skeletal system which was pointed out to impact muscle force distribution [24]. Active muscle structures modeled as muscle elements were assumed to act along straight lines neglecting volumetric effects [45]. Besides bone wrapping, additional segment-fixed via-points were incorporated to gain a more realistic representation of curved muscle paths especially adjacent to the hip joint, suggested to improve predictions of reaction force components [78]. This muscle discretization, however, may not entirely reflect physiological deflections of muscle paths along with complex intermuscular contact interactions, which in particular applies on muscles with large curvatures. Further improvements in this regard may be obtained by implementing via-points movable along wrapping surfaces [70, 78], with verification against muscle paths derived from MRI data [68]. According to Vrahas et al. [79] passive soft tissue contribute less than 10% to intersegmental torques during gait and stair climbing. Consequently, passive forces arising from muscular or capsular structures were not considered. While this simplification may hold true for common activities, the effect of these structures should be reevaluated for extreme dislocation-associated maneuvers.

Estimation of muscle forces was based on an inverse dynamics analysis in order to gather hip joint reaction forces. The current model formulation comprises two redundancy problems. Vaughan et al. [46] indicated that the the neuromuscular strategy may be based on minimizing joint torques during activities with at least one loop closure. As their optimization approach avoids computationally expensive calculations of explicit loop closure conditions [37], a similar way was pursued in the present work. To further reduce modeling complexity, the closed loop problem was accounted for by defining symmetry conditions with respect to the pelvic sagittal plane omitting the contralateral limb. This simplification is supported by findings in THR patients indicating equivalent kinematics between operated and non-operated hip and no overcompensation by the contralateral side during sitting down and standing up activities [80]. However, the symmetry conditions should be reconsidered when other maneuvers are the subject of interest.

The distribution problem of muscle forces was addressed by using optimization techniques [44, 45]. Although it seems appealing that the nervous system governs motion by controlling muscle forces in an optimal manner, it remains an intricate task to find physiological or even neurophysiological evidence of appropriate cost functions. Both synergistic and antagonistic activity comparable to EMG data were found to be estimated when using non-linear cost functions in contrast to linear ones [81, 82]. Other factors such as the definition of weighting coefficients may also promote antagonistic prediction [83]. However, Herzog and Binding [84] showed that co-contraction is only predicted when multi-joint antagonists are present. Independent from the definition of the cost functions, it was also observed that muscle force prediction reacts with great sensitivity to model-based deviations of kinematic data [85], muscle origin and insertion points [86], lever arms and physiological cross section areas [87]. Furthermore, constraining muscle forces within physiological boundaries was shown to remarkably reduce the number of possible solutions [88]. These findings support the notion that detailed modeling of the musculoskeletal system rather leads to realistic results than the optimization procedure per se. Hence, an approach similar to Anderson and Pandy [40] was followed in this work, which allowed incorporation of muscle architecture and physiology explicitly in the equations of motion.

Despite the limitations involved with musculoskeletal modeling, Brand et al. [73] reported comparable peak predictions and patterns during gait against hip contact forces arising from one patient with an instrumented implant. Heller et al. [55] used a cycle-to-cycle comparison to validate predicted hip joint reaction forces against in vivo data from four patients, revealing good agreement in both patterns and magnitudes. Their calculated peak forces overestimated the measurements with deviations ranging from 0.3% to 33% for walking and from 3% to 37% during stair climbing. In this sense, the HiL test system revealed physiologically reasonable force levels, with deviations at absolute peak values of 10.6% to 26.2% for normal sitting down and 4.9 to 21.5% for normal standing up, respectively, against in vivo data of three patients [33]. Analogous to Martelli et al. [68], further differences noted within this work may arise from diverging health conditions of the modeled subject and the instrumented patients [33], as well as varying implant positions [23]. Moreover, Nadzadi et al. [18] estimated considerably elevated hip joint reaction forces for extreme sit-to-stand maneuvers based on a previously validated model [73], consistent with the HiL outcomes of the dislocation-associated maneuver. Hence, it can be inferred that the HiL test system is capable of reproducing physiological conditions for testing THR dislocation; at least for symmetric leg maneuvers.

Depending on the surgical approach specific muscular and capsular structures are incised, resected or damaged intraoperatively to gain access to the hip joint. Especially, the posterior approach involves larger loss of soft tissue as it requires resection of the small external rotators as well as incision of the posterior capsule [65]. Hence, it seems not surprising that this approach was reported to entail higher dislocation rates than others [29]. Pellicci et al. [27] argued that these unsatisfactory outcomes are caused by the dead space, left after resected soft tissue and usually found posteriorly in revision procedures. By performing enhanced soft tissue repair before closure, they succeeded to significantly improve the postoperative results as confirmed subsequently by other clinicians [28, 29].

Although the mechanical aspects of the hip capsule have been illuminated to a certain extent [89–91], there are only few studies providing insights in how soft tissue structures contribute to resistance against THR dislocation. Delp et al. [26] stated that extension of the femoral offset, a widespread medium by surgeons to adjust tension on the hip joint, increases the muscles’ active moment-generation capacity and passive muscular forces. Specimen-based studies [30, 31] indicated that full repair of muscle and capsule tissue after the posterior approach lead to augmentation of the torque measured along internal/external rotation until final dislocation in contrast to no or minor repairs. Elkins et al. [32] concluded that well-designed repairs are able to restore integrity of capsular structures which was proven by similar resisting torques against dislocation as obtained for the intact capsule. The findings of this work suggest that resection of the small external hip rotators may increase the risk of dislocation due to decrease in load level and inauspicious alterations of force directions, in particular for malpositioned implants. Furthermore, the mentioned muscle structures seem to provide active resistance against the dislocation process when remained intact. These suggestions, however, need to be substantiated further within subsequent studies. This includes incorporating the mechanical response of the hip capsule to clarify to what extend these structures mitigate dislocation.

The presented validation and first research applications show that HiL simulations are able to predict the influence of cup positioning and muscle removal as well as lubrication and body mass on THR stability. In future studies, further parameter sets regarding implant designs and positions will be tested in order to gain new insights into impingement and dislocation processes which improve implant safety as well as surgical technique.
